# HIV epidemic in Lesotho – HIV treatment and monitoring of patients on antiretroviral therapy 2014-2024

**DOI:** 10.1371/journal.pgph.0006499

**Published:** 2026-07-27

**Authors:** Senate Moshoeshoe, Maletsatsi Motebang, Kamohelo Mokhesi, Mamorapeli Ts’oeu, Puleng Ramphalla, Yohannes Mengistu, Shirley Lecher

**Affiliations:** 1 Division of Global HIV and Tuberculosis, Center for Global Health, Centers for Disease Control and Prevention, Maseru, Lesotho; 2 Global Health International, Association of Public Health Laboratories (APHL), Addis Ababa, Ethiopia; 3 Division of Global HIV and Tuberculosis, Center for Global Health, Centers for Disease Control and Prevention, Atlanta, Georgia, United States of America; University of Ottawa, CANADA

## Abstract

This study evaluates progress in expanding access to antiretroviral therapy (ART) and HIV viral load (VL) monitoring in Lesotho using programmatic data 2014–2024. As a lower middle-income country in Southern Africa, Lesotho maintains the world’s second highest HIV prevalence (25.6%).Retrospective, countrywide data from 2014-2024 were assessed, including the cumulative number of people living with HIV receiving ART, the proportion of ART patients who had a VL test within the prior 12 months, the percentage of VL tests indicating viral suppression (<1,000 copies/mL) and the turnaround time (TAT) from specimen collection to result return.From 2014 to 2024, the number of PLHIV on ART more than doubled, rising from 111,322–242,438. VL coverage progressed from 6% in 2014 to 93% in 2024. VL suppression improved from 75% in 2015 to 99% in 2024. Mean TAT declined from 56 to 8 days over the study period.Lesotho achieved substantial expansion of ART coverage, VL testing, and timely result reporting during the study period 2014–2024. This report documents Lesotho’s achievement to reach the third UNAIDS “95” goal that 95% of persons on ART are virally suppressed. These findings underscore the feasibility of scaling up HIV service delivery, achieving high levels of viral suppression and strengthening laboratory systems in high-prevalence, resource limited countries.

## Author summary

As the number of PLHIV on ART increases, from 2014 to 2024 efforts to strengthen monitoring led to increase in HIV viral load coverage and suppression. there was a significant increase in testing statics and decrease in Turnaround time.

## Introduction

Southern Africa remains disproportionately affected by HIV and represents more than 50% of all new HIV infections [1–3]. Lesotho is a lower middle-income country in southern Africa with the world’s second highest HIV prevalence [4]. According to a 2020 HIV Population-based Impact Assessment, the overall HIV prevalence rate in Lesotho was 25.6% [5]. This heavy burden of HIV has threatened the country’s stability and future economic success. Persons living with HIV (PLHIV) now have extended life expectancy due to over two decades of investments through PEPFAR, the Global Fund and other international partners in HIV testing, antiretroviral treatment (ART) and monitoring to verify durable viral suppression which is required to preserve health and prevent further HIV transmission [6]. Consistent ART use decreases morbidity and mortality in PLHIV, prevents vertical transmission of HIV and is effective in preventing sexual transmission when viral load is suppressed [7–9]. The Joint United Nations Programme on HIV/AIDS (UNAIDS) has set “95-95-95” targets for HIV infection control by 2030 to: 1) increase to 95% the proportion of people living with HIV who are aware of their HIV status, 2) ensure that 95% of these individuals receive ART and 3) achieve viral load testing and suppression (viral load ≤1,000 HIV RNA copies/mL of blood) among 95% of those receiving ART [10]. In efforts to control the HIV epidemic, Lesotho intensely focused on increasing ART access and viral load monitoring through increasing molecular diagnostic laboratory capacity after the launch of “Test and Treat” for all PLHIV, adopted from the World Health Organization (WHO) 2016 recommendations [11]. As ART access increased, scaling-up viral load testing to monitor viral suppression became a parallel priority for the government of Lesotho and has been established as a global health priority [12]. The government of Lesotho, in partnership with PEPFAR, the Global Fund, UNAIDS and other international partners, developed multiple collaborative initiatives to accelerate progress towards HIV Epidemic control. This report documents the achievements from 2014 through 2024 in increasing ART access for PLHIV and scaling up HIV viral load testing for all patients receiving ART in Lesotho.

## Methods

Programmatic data were collected between January 2018 through December 2024 from standardized Monitoring Evaluation and Reporting (MER) programmatic datasets, using the MER defined indicators for coverage and suppression. MER data are collected routinely by quarter for all PEPFAR supported countries and publicly available through the PEPFAR Panorama data platform data.pepfar.gov/datasets. PEPFAR supports all 10 districts in Lesotho. PEPFAR data are considered nationally representative and reported through health care facilities and the laboratory information system to the Ministry of Health. All data used in this analysis are provided in this study. CDC program officers collected information on cumulative number of patients receiving ART, viral load coverage defined as the number of ART patients eligible for a viral load test in the past 12 months who received ≥ 1 viral load test result, viral load suppression defined as the proportion of persons who received a viral load test <1,000 HIV RNA copies/mL of blood and the mean turnaround time (TAT). The mean TAT was defined as the average time (in days) from sample collection to the time viral load results were returned from the testing laboratory to the collecting facility. The number of results with viral suppression was calculated by dividing the number of viral load tests with <1000 copies/mL by total number of viral load test results. National guidelines indicate a viral load test 6 months after ART initiation, followed by testing 12 months and annually thereafter for adults. Pregnant and breastfeeding women and children are monitored every 6 months. ART expansion occurred as the Lesotho national guidelines were updated to reflect WHO guidance to treat all persons diagnosed and living with HIV [11]. As more effective regimens became available, ART regimens changed to include integrase inhibitors. PEPFAR investments supported the cost for scaling up ART and viral load testing.

The scale-up of viral load testing began in 2014 starting with one laboratory which initially targeted only patients with clinically suspected failure. In 2015, Lesotho implemented the National Strategy for scaling up HIV viral load, which required a shift from targeted to routine viral load testing for all PLHIV on ART. Laboratory infrastructure rapidly expanded to include seven laboratories from 2014 through 2018 [13]. Laboratory hubs were also established to improve testing efficiency and decrease the TAT from specimen collection to viral load testing.

This activity was reviewed and approved in accordance with the U.S. Centers for Disease Control and Prevention (CDC) human research protection procedures and was determined to be non-research consistent with CDC policy and applicable federal law. Applicable federal law for ethical review include: 45 C.F.R. part 46.102(l)(2), 21 C.F.R. part 56.

## Results

Between 2014 and 2016, there was a 57 percent increase in number of patients on ART (Table 1). From 2016 to 2020 the number of patients on ART continued to increase and additional 33% from 175,272–232,304. There was a slight reduction in 2021 likely due to interruptions in services due to the COVID-19 pandemic. Despite this reduction, the number of patients on ART from 2014 to 2024 showed 118% growth over the period. This is a major expansion in a decade (Table 1).

**Table 1:** Number of patients on ART, Number of patients with more than 1 viral load tests and percentage of suppression and TAT for VL results from 2014 to 2024.

	Total Number of ART patients	Number of Patients with ≥1 Viral Load test	TAT (in days)	% of VL tests with viral suppression (<1000 cp/ml)#
2014	111,322	6,391	56	**
2015	129,127	11,265	28	75%
2016	175,272	22,996	28	87%
2017	201,758	66,296	56	90%
2018	218,493	113,540	28	93%
2019	226,590	179,679	28	95%
2020	232,304	195,130	23	97%
2021	232,182	204,145	16	98%
2022	233,736	212,328	14	98%
2023	240,540	218,878	12.3	99%
2024	242,438	223,819	8.1	99%

**Data unavailable.

In 2014, only 6,391 patients received a viral load test, representing 6% of patients on ART (Table 1). With the expansion of laboratory capacity there was a 57% increase in the number of persons receiving a viral load test by 2016. As the number of ART patients increased, the demand for viral load testing increased, resulting in a nearly tenfold increase in the number of persons receiving a viral load test from 2016 to 2024 (Table 1). By 2024, 92% of patients on ART received a viral load test.

TAT for viral load testing improved considerably from 56 days in 2014–8 days in 2024. The 2017 TAT increase noted was due to frequent equipment breakdowns, leading to specimen backlogging. Once these issues were resolved, the TAT decreased. By 2021, the average TAT was 16 days which equates to a 71% reduction in TAT, with a further reduction to 8 days in 2024. (Table 1).

**Fig 1:**
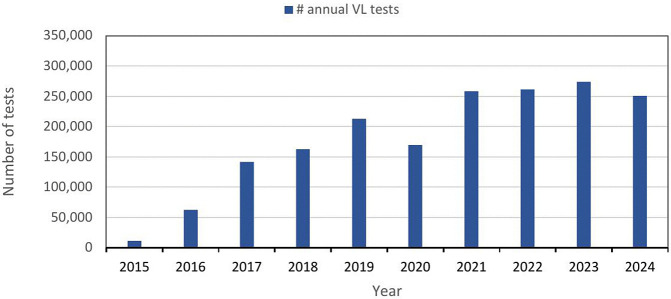
Annual number of VL test performed from 2015 to 2024.

Progression in capacity for viral load testing reflected in the annual number of VL tests performed 2015–2024 (Fig 1). Testing volumes reflect testing for all populations including pregnant women and children who may have received more than one test.

**Fig 2:**
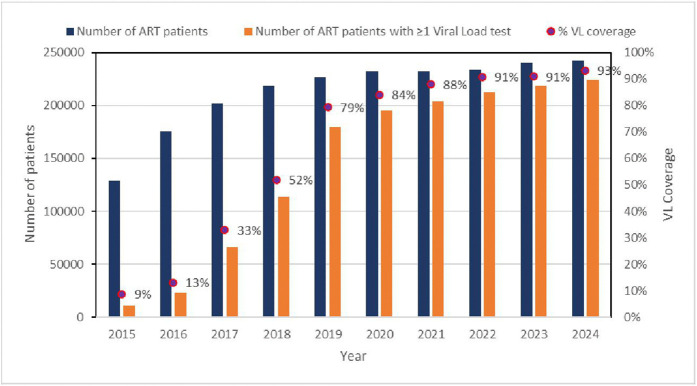
Viral load coverage on ART patients from 2015 to 2024.

Overall, viral load coverage for patients on ART gradually increased from 6% in 2014 (Table 1), 9% in 2015 to 79% in 2019 (Fig 2). From 2014 to 2016: growth is real but coverage remains low (6% to 13%). This aligns with early-stage capacity building and initially targeted testing. From 2017 to 2019 there is rapid scale-up (coverage roughly 33% to 79%). This likely reflects the shift to routine VL monitoring plus major lab capacity expansion and system strengthening. As of 2020–2024 coverage rises further into the low 90s and stabilizes (84% in 2020 to 92% in 2024). Viral load coverage increased to 93% by 2024. Viral load coverage was calculated using the MER indicator definition of “ART patients eligible for a VL test in the past 12 months who received ≥1 VL result.”

**Fig 3:**
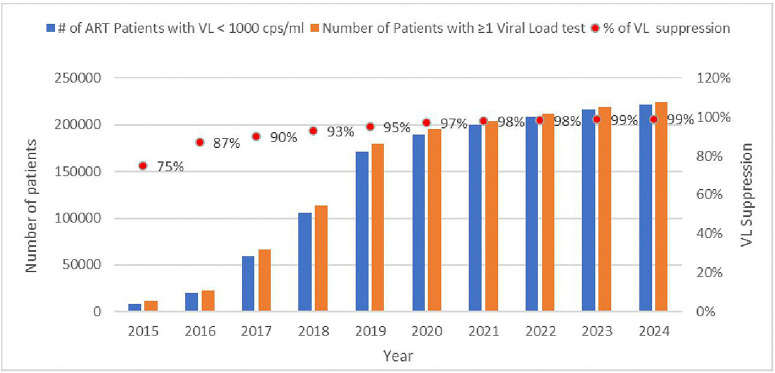
Viral load suppression on ART patients from 2015 to 2024.

In line with ART expansion, improvements in ART regimens and viral load monitoring strategies, viral load suppression improved substantially. The national viral load suppression rate increased from 75% in 2015 to 99% in 2024 (Fig 3).

## Discussion

Lesotho has made sustained progress over the past decade toward achieving the “95-95-95” goals (27,28). During the period of ART and viral load scale-up from 2014 through 2024, Lesotho successfully doubled the number of patients on ART. The early surge is consistent with policy/strategy effects (e.g., “Test and Treat” adoption and active scale-up). The later plateau may reflect reaching saturation among diagnosed PLHIV, slower net new enrollment due to stable coverage, population dynamics, retention challenges, or limits in identifying untreated PLHIV.

Major laboratory system strengthening led to notable TAT improving rapidly to 28 days between 2015 and 2016, however spike back to 56 days attributed to equipment breakdowns and backlogs. Nevertheless, a steady improvement was seen from 2018 to 2024 reaching 16 days in 2012 and 8 days by 2024. The 2017 spike is operationally informative: it highlights that scale-up creates vulnerability unless maintenance, supply chains, redundancy, and service contracts are robust. The long-run decline to 8 days signals a mature laboratory network: improved capacity, improved logistics, improved workflow, and likely better data systems (lab information systems and communication).

Targets were achieved in increasing the number of PLHIV on ART and viral load testing. Viral load coverage increased to 93% while maintaining VL suppression of 99% for two years 2023 and 2024. VL monitoring shifted from very limited to near-universal, which is one of the most important program achievements. Even though 92–93% coverage achieved in this report is extremely strong, the final step to consistent >95% annual coverage is usually the hardest: it requires tightening systems for the most mobile, underserved, or least engaged patients.

Like many countries, the COVID-19 pandemic created challenges, and southern Africa was more severely impacted than other areas of sub-Saharan Africa [14–16]. Improvements in ART access and viral load coverage occurred through program adaptation and resilience, to successfully overcome supply chain shortages and service disruptions experienced in many countries. While access to ART has improved, new challenges emerged, including long-term adherence, retention in care, improving access to viral load testing in rural areas and vulnerable populations including infants and pregnant women [17,18]. PEPFAR supports patient-centered differentiated service delivery as an important and potentially impactful intervention, along with other evidence-based methods such as expanding access to multi-month dispensing to support lifelong continuation of treatment [19]. Health system strengthening was required for ART distribution, investment in human capacity building, removal of barriers to services, and implementation of continuous quality improvement measures. Through donor investments and technical assistance, substantial progress was achieved including expansion of the sample referral network, training in molecular diagnostics, developing a quality assurance program, building information systems for data capture and supporting surveillance and results dissemination. Testing capacity was expanded by developing hubs throughout the country using a combination of high throughput and point-of-care molecular technology. For remote and hard-to-reach rural areas, motorbikes serve as the primary means of sample transportation, horses and small airplanes are used in some destinations where access is limited. Technology innovations, including the implementation of an electronic registry for health care facilities, diagnostic network optimization and the development of a national dashboard for testing results helped decrease the TAT and transmit data to the Ministry of Health to inform timely decisions. Lesotho appears to have moved from “expansion” to “optimization”. Between 2014–2016. There was focus to build on and expand access. There was rapid ART enrolment growth, initial VL capacity and early gains in TAT. By 2017–2018, VL scale up was evident. Monitoring was national, coverage climbed sharply and suppression improved and crossed 90% to 95%. The 2020–2024 period demonstrates a consolidate, improved performance and mature system. This is noted by ART numbers plateau, VL coverage reaching >90%, TAT improvements to near operationally responsive timeframes and suppression stabilizing at high levels of 98% to 99%.

The findings in this report are subject to at least two limitations. Data sources could not exclude the possibility of patient-level duplication as unique patient identifiers are not available in Lesotho. Patients could potentially go to more than one facility without the results being linked. Thus viral suppression is calculated at the test level. Nevertheless, the analysis is described as countrywide and drawn from standardized PEPFAR MER datasets, with coverage across all 10 districts. That is valuable because it reflects real program performance, not a small study cohort.

Viral suppression was defined as a viral load ≤1,000 HIV RNA copies/ml, which does not account for patients with low level viremia <999 copies/ml.

Lesotho’s progress in advancing HIV treatment and viral load testing between 2014 and 2024 demonstrated steady advancement to achieve the 2nd and 3rd “95” targets for HIV epidemic control [20,21]. The program data in this report align with improvement in round one of the Lesotho Population Based HIV Impact Assessment (PHIA) 2016–2017 reporting 91.8% of people living with HIV on ART and viral load suppression of 87.7% and the second round PHIA completed in 2020 [22]. According to the second Lesotho PHIA 2020 report, 90.1% of adults living with HIV were aware of their status, 96.9% of them were on ART, of whom 91.5% had suppressed viral loads [5]. Till then progress has been made with Lesotho demonstrating 95% for awareness, 94% for treatment and 99% suppression [23]. To fully reach the 95-95-95 targets, the use of evidence-based and innovative strategies to overcome existing challenges is essential [24,25]. While the observed trends are encouraging, the scale up of high-quality testing services and treatment strategies will remain indispensable.

Several questions that remain outside the scope of this report include Incidence and transmission impact, patient group disparities, Retention and loss to follow-up and Clinical action after VL results. What remains credible from the research data is the direction of change due to ART and VL monitoring, system improvement and suppression improvement.

## Conclusions

Achievements in Lesotho toward the “95” UNAIDS goals over the past decade have been documented in this report. Lesotho serves as a success story for donor investments addressing the HIV epidemic in low- and middle-income countries. Key factors contributing to ART expansion and viral load testing include increased access to health care facilities and laboratory testing for PLHIV, partnerships with international donors, strong government support, and optimization of clinical and laboratory systems. Drawing on key lessons from the past decade, Lesotho is well positioned to maintain a trajectory toward meeting the ultimate goal of ending the AIDS epidemic by 2030.

The findings and conclusions in this report are those of the author(s) and do not necessarily represent the official positions of the funding agencies. This report was supported by the President’s Emergency Plan for AIDS Relief (PEPFAR) through the Centers for Disease Control and Prevention (CDC).
